# Revisiting viral reprogramming: Measles virus as a robust platform for high-quality iPSC generation

**DOI:** 10.1016/j.omta.2026.201759

**Published:** 2026-05-22

**Authors:** Mohamed Taha Moutaoufik, Mohamed Chahine

**Affiliations:** 1 Faculty of Medical Sciences, UM6P Hospitals, Mohammed VI Polytechnic University, Ben Guerir, Morocco; 2 CERVO Brain Research Centre, Quebec City, QC, Canada; 3 Department of Medicine, Faculty of Medicine, Laval University, Quebec City, QC, Canada

## Main text

Induced pluripotent stem cells (iPSCs) have become a powerful tool for modeling human disease *in vitro*, particularly for investigating patient-specific genetic disorders. By reprogramming somatic cells into pluripotent cells and differentiating them into relevant lineages, such as cardiomyocytes, neurons, or skeletal muscle cells, it is possible to reproduce key cellular and functional disease phenotypes under controlled conditions.

One of the most common reprogramming systems relies on vectors derived from Sendai virus (SeV). These vectors do not integrate into the genome and exhibit high reprogramming efficiency, enabling robust acquisition of the pluripotent state, which makes this strategy particularly attractive for clinical applications. The utilization of Yamanaka factors, OCT4, SOX2, KLF4, and c-MYC guarantees stable expression of the necessary genes and a low risk of insertional mutagenesis in comparison with the usage of integrative retroviral and lentiviral vectors.^1^ Nonetheless, some concerns regarding the presence of residual viral RNA and differences between individual cell lines persist.

Regarding this problem, in this issue of *Molecular Therapy Advances*, the team of researchers describes the measles virus (MeV) vector-based reprogramming system that also operates on the principles of cytoplasmic replication and therefore does not cause insertion of additional genetic material.^2^

As far as one can conclude based on the results described, this system demonstrates good efficiency of reprogramming factor expression and may prove valuable for further investigation as an alternative to the SeV system.

Regarding the mentioned modification of the MeV-based reprogramming system, the authors present MVPWT(O) (SK) (M), which was designed through replacement of the P gene coding for the phosphoprotein of the Moraten backbone by that of the wild type Ichinose-B strain of MeV. Since the P protein takes part in the formation of paramyxovirus polymerase along with the nucleoprotein N and L protein, its substitution should have a certain effect on transcription and replication. One more feature of this system is the usage of green fluorescent protein to monitor the infection progress and optimize MOI. According to the obtained results, there is evidence that the developed vector maintains the characteristics of growth kinetics and titers (∼10^6^ per 48 h). At that, the reduced fitness of the virus due to genome manipulation should affect the reprogramming efficiency adversely.

### Enhanced reprogramming efficiency and robustness

A marked increase in reprogramming efficiency is observed compared to the previous MeV vector. The reported range (0.23%–2.25%) corresponds to an approximately 50-fold improvement over the earlier 0.04% value.^3^^,^^4^ Within the context of reprogramming systems, this brings the MeV platform into a similar range as established approaches such as SeV (See Table 1 comparing the performance of the two systems). Optimization of the MOI reveals a balanced efficiency between transduction and cytotoxicity. Although higher MOIs (1–2) enhanced early transduction, they were associated with toxicity by day 3, likely reflecting increased viral replication and cellular stress. An MOI of 0.5 appears to provide a suitable compromise, allowing effective delivery of reprogramming factors while limiting cell loss. This observation highlights that higher transduction levels do not necessarily translate into improved reprogramming outcomes. The MVPWT(O) (SK) (M) vector was able to reprogram all tested adult human fibroblast (AHF) lines, including AHF4, which was not successfully reprogrammed using SeV. This suggests that the MeV-based system may be less sensitive to donor-dependent variability, potentially related to differences in antiviral responses, epigenetic state, or cell cycle dynamics. Such robustness across donor variability is a major advantage for translational and personalized medicine applications.

**Table 1:** Comparison of MeV and SeV reprogramming systems

Feature	measles virus (MeV)	Sendai virus (SeV)
Virus type	paramyxovirus (negative-sense RNA)	paramyxovirus (negative-sense RNA)
Genome integration	non-integrating (cytoplasmic replication)	non-integrating (cytoplasmic replication)
Reprogramming efficiency	high; can be optimized	very high; well established
Expression of factors	strong and adjustable	robust and controlled
Clearance	passage-dependent can be engineered	efficient (temperature-sensitive or dilution through proliferation)
Clinical track record	emerging	established
Vector flexibility	decent	moderate
Pluripotent capacity	maintains full pluripotency.	maintains full pluripotency
Differentiation capability	validated in some lineages	established in multiple lineages
Safety profile	non-integrating; require validation	non-integrating well established
Scalability	potential for scale-up	high, scalable

### Clone stability and reprogramming fidelity

When comparing iPSCs generated using the two vectors (MeV and SeV), differences in the stability of the reprogramming process can be observed. MeV-derived clones were maintained long term and retained stable growth characteristics up to passage 40, whereas some SeV-derived iPSC clones displayed reduced stability and signs of spontaneous differentiation during extended culture. This difference can be due to certain peculiarities in delivery of reprogramming factors via these vectors. Incomplete reprogramming of donor somatic cells is often a problem when generating iPSCs, which usually results in colony instability as a result of incomplete reversal of the epigenetic state. It is worth noting that the stability of MeV-derived iPSC clones can be due to higher efficiency in inducing pluripotency; however, this requires further research and direct comparison. Finally, the use of several clones generated from independent reprogramming events increases the reliability of the results, and the consistent stability of iPSC colonies across multiple donor lines supports the robustness and reproducibility of the MeV system.

### Rapid and efficient vector clearance

Non-integrating vectors are preferable in iPSC generation since they allow losing extraneous sequences after induction of pluripotency. As was mentioned previously, the majority of MeV-derived iPSCs became free of vector sequences by passage 5, with the decrease starting already by passage 4. Such rapid removal can be attributed to low MOI and cell division; moreover, this efficiency was confirmed by the absence of contamination in experiments performed by other authors. Detection of MeV sequences was conducted via quantitative PCR targeting nucleocapsid gene; the detection limit of the method was claimed to be only four copies, which provides very accurate monitoring of the vector presence. Thus, from the application point of view, the efficiency of vector removal is very important since persistent viral nucleic acid may affect genomic stability or induce cellular responses. The tested MeV system proves to work efficiently at least in this case.

### Pluripotency and differentiation capacity

According to the data obtained, all MeV-derived iPSCs had characteristics of normal pluripotent stem cells; thus, they expressed OCT4, SOX2, NANOG, SSEA 4, TRA 1 60, and TRA 1 81 genes. Moreover, there were no significant differences between iPSCs derived from male and female donors. Finally, all iPSC clones formed embryoid bodies and differentiated into the three germ layers. Variability in spontaneous endoderm differentiation was noted for certain clones; however, this is usual for the iPSC population. Finally, successful directed differentiation of endodermal derivatives in all tested iPSC clones is crucial for confirming proper functionality of MeV-derived iPSCs. Differentiation into neuronal, pancreatic, and hematopoietic progenitors further supports the functional competence of these iPSCs. Hematopoietic differentiation was particularly efficient, with CD34^+^/CD45^+^ and CD34^+^/CD43^+^ populations exceeding manufacturer-reported benchmarks. Although these results demonstrate robust hematopoietic output, they do not, on their own, support a conclusion of enhanced mesodermal differentiation capacity. Further systematic comparative studies across mesodermal lineages would be required to determine whether MeV-derived iPSCs exhibit broader lineage-specific advantages relevant to disease modeling and regenerative applications.

The generation of colony-forming units (CFUs) confirms that the hematopoietic progenitors are functionally active not merely defined by surface markers. This level of validation strengthens the utility of these iPSCs for downstream research applications.

### Genomic stability and identity

Genomic integrity is a key criterion for assessing iPSC quality, especially in contexts aimed at clinical use. Nineteen of the twenty MeV-derived clones retained a normal diploid karyotype through passage 25, indicating stable chromosomal maintenance during expansion. The single clone showing a partial 18q deletion in a small subset of cells, most probably an isolated event rather than a pattern associated with the reprogramming approach; however, this finding should be noted and warrants careful consideration and further monitoring to exclude culture-associated or selection-related genomic changes. Short tandem repeat (STR) fingerprinting confirmed that each clone matched its parental fibroblast line, ruling out cross-contamination or sample mix-ups. All cultures also tested negative for mycoplasma, supporting the reliability of subsequent functional analyses. Overall, these findings suggest that the MeV-based reprogramming system does not introduce overt genomic abnormalities as assessed by the available karyotype and STR analyses, in line with expectations for a non-integrating viral vector system. However, a more comprehensive assessment of genomic integrity, such as SNP array, copy-number variation (CNV) profiling, whole-genome sequencing, and extended passage monitoring, would be required to fully exclude subtle genomic alterations.

### Conclusion

The MeV system improves iPSC technology by combining efficient reprogramming, safety, and flexibility, thereby addressing several limitations of current methods. Importantly, as a promising and technically flexible non-integrating platform, the MeV system now warrants rigorous head-to-head benchmarking against SeV and other established non-integrating methods to fully establish its relative performance, scalability, and translational potential. Important caveats should be addressed. In particular, aspects related to GMP readiness, regulatory familiarity, biosafety considerations, and potential translational barriers compared with established SeV-based approaches are warranted.

## Acknowledgments

This work was supported by a U.S. Department of Defense grant (USAMRAA W81XWH-21-1-0426) and two Canadian Institutes of Health Research grants (MOP-111072 and MOP-130373) to M.C.

## Declaration of interests

The authors declare no competing interests.
